# Accounting for sex differences in PTSD: A multi-variable mediation model

**DOI:** 10.3402/ejpt.v6.26068

**Published:** 2015-01-19

**Authors:** Dorte M. Christiansen, Maj Hansen

**Affiliations:** 1 Department of Psychology, University of Aarhus, Aarhus C, Demark; 2 National Centre for Psychotraumatology, University of Southern Denmark, Odense M, Denmark; 3 Institute of Psychology, University of Southern Denmark, Odense M, Denmark

**Keywords:** Posttraumatic stress disorder, gender differences, multiple mediators, risk factors, predictors, robbery, interpersonal violence

## Abstract

**Background:**

Approximately twice as many females as males are diagnosed with posttraumatic stress disorder (PTSD). However, little is known about why females report more PTSD symptoms than males. Prior studies have generally focused on few potential mediators at a time and have often used methods that were not ideally suited to test for mediation effects. Prior research has identified a number of individual risk factors that may contribute to sex differences in PTSD severity, although these cannot fully account for the increased symptom levels in females when examined individually.

**Objective:**

The present study is the first to systematically test the hypothesis that a combination of pre-, peri-, and posttraumatic risk factors more prevalent in females can account for sex differences in PTSD severity.

**Method:**

The study was a quasi-prospective questionnaire survey assessing PTSD and related variables in 73.3% of all Danish bank employees exposed to bank robbery during the period from April 2010 to April 2011. Participants filled out questionnaires 1 week (T_1_, N=450) and 6 months after the robbery (T_2_, N=368; 61.1% females). Mediation was examined using an analysis designed specifically to test a multiple mediator model.

**Results:**

Females reported more PTSD symptoms than males and higher levels of neuroticism, depression, physical anxiety sensitivity, peritraumatic fear, horror, and helplessness (the A_2_ criterion), tonic immobility, panic, dissociation, negative posttraumatic cognitions about self and the world, and feeling let down. These variables were included in the model as potential mediators. The combination of risk factors significantly mediated the association between sex and PTSD severity, accounting for 83% of the association.

**Conclusions:**

The findings suggest that females report more PTSD symptoms because they experience higher levels of associated risk factors. The results are relevant to other trauma populations and to other trauma-related psychiatric disorders more prevalent in females, such as depression and anxiety.

Females develop posttraumatic stress disorder (PTSD) about twice as often as males (Christiansen & Elklit, 2012; Kessler, Sonnega, Bromet, Hughes, & Nelson, 1995; Tolin & Foa, 2008). Sex differences in PTSD are not simply a product of measurement error, methodological bias, or reporting bias, but appear to reflect substantive differences (Christiansen & Elklit, 2012; Chung & Breslau, 2008; Tolin & Foa, 2008). Both biological sex and gender-related concepts such as gender-role orientation and sexuality are likely to affect sex differences in PTSD. The mediation hypothesis suggests that sex differences in PTSD are related to differences in associated risk factors in the time prior to, during, and following the trauma (Christiansen & Elklit, 2012). Thus, risk factors that are more prevalent in females may account for sex differences in PTSD severity.

## Pretraumatic risk factors

Males generally report more traumatic exposures (Kessler et al., 1995; Tolin & Foa, 2008), but females are exposed to more sexual trauma, a particularly toxic trauma type (Tolin & Foa, 2008). As sex differences exist across trauma types and after individuals with prior exposure to sexual trauma have been cancelled out, researchers have concluded that the increased exposure of females to sexual trauma cannot fully account for sex differences in PTSD (Kessler et al., 1995; Olff, Langeland, Draijer, & Gersons, 2007; Stein, Walker, & Forde, 2000; Tolin & Foa, 2008). However, such studies do not take prior sexual trauma into account. Thus, the full impact of sexual trauma on sex differences in PTSD is unknown. Females also report more depression (Olff et al., 2007), neuroticism/negative affectivity (Breslau, Davis, Andreski, Peterson, & Schultz, 1997; Christiansen & Elklit, 2012; Zeidner, 2006), and anxiety sensitivity (Stewart, Taylor, & Baker, 1997), particularly physical anxiety sensitivity, than males (Lang, Kennedy, & Stein, 2002; Stewart et al., 1997). As these variables have all been associated with higher levels of PTSD severity (DiGangi et al., 2013; Lang et al., 2002; Nillni, Berenz, Pineles, Coffey, & Zvolensky, 2013; Olff et al., 2007; Taylor, Koch, & McNally, 1992), they may help account for sex differences in PTSD. However, to the best of our knowledge, anxiety sensitivity has not been examined as a potential mediator, and sex differences in neuroticism and depression cannot fully account for sex differences in PTSD (Christiansen & Elklit, 2012; Olff et al., 2007).

## Peritraumatic risk factors

Even though the A_2_ criterion has been removed from the DSM-5 (American Psychiatric Association, 2013), peritraumatic emotional responses such as intense fear, horror, and helplessness remain important risk factors for PTSD (Bovin & Marx, 2011; Pacella et al., 2011). Other peritraumatic reactions that predict PTSD include dissociation (Breh & Seidler, 2007; Olff et al., 2007; Ozer, Best, Lipsey, & Weiss, 2003), panic (Boscarino & Adams, 2009; Bryant et al., 2011; Lawyer et al., 2006; Rocha-Rega et al., 2009), and tonic immobility (Abrams, Carleton, Taylor, & Asmundson, 2009; Lima et al., 2010; Rocha-Rega et al., 2009). All except for tonic immobility have been reported to be more common in females than males. (Boscarino & Adams, 2009; Breslau & Kessler, 2001; Christiansen & Elklit, 2012; Lawyer et al., 2006; Olff et al., 2007). Although tonic immobility is not unique to sexual trauma (Bados, Toribio, & García-Grau, 2008), it has been mostly studied in females. Two studies have examined and failed to find sex differences in tonic immobility (Abrams et al., 2009; Abrams Carleton, & Asmundson, 2011). However, as both studies were based on very small, predominantly female samples, they may have lacked sufficient statistical power to detect sex differences. Thus, in spite of prior findings we expect females to report more tonic immobility than males. It has been suggested that the origin of sex differences in PTSD may be closely linked to sex differences in the subjective experience and evaluation of the trauma, rather than more objective features, such as trauma type and degree of exposure (Spindler, Elklit, & Christiansen, 2010). Thus, these peritraumatic reactions may all help account for sex differences in PTSD. We are not aware of any studies that have examined peritraumatic panic or tonic immobility as potential mediators between sex and PTSD, but neither dissociation nor fear, horror, and helplessness can fully account for sex differences in PTSD (Christiansen & Elklit, 2012; Olff et al., 2007).

## Posttraumatic risk factors

Lack of posttraumatic social support is a strong risk factor for PTSD (Brewin, Andrews, & Valentine, 2000). Negative social support or feeling let down by others is generally found to be a better predictor of PTSD than positive social support (Christiansen & Elklit, 2008). Similarly, negative posttraumatic cognitions are thought to produce an on-going sense of threat that is essential to PTSD (Ehlers & Clark, 2000). Negative posttraumatic cognitions concerning self, the world, or self-blame have been reported to be strongly associated with PTSD severity (Blain, Galovski, Elwood, & Meriac, 2013; Foa, Ehlers, Clark, Tolin, & Orsillo, 1999; Moser, Hajcak, Simons, & Foa, 2007), although for self-blame the association is not always positive (Blain et al., 2013; Daie-Gabai, Aderka, Allon-Schindel, Foa, & Gilboa-Schechtman, 2011; Moser et al., 2007; O'Donnell, Elliott, Wolfgang, & Creamer, 2007). Females generally report more negative social support (Andrews, Brewin, & Rose, 2003) and negative cognitions (Tolin & Foa, 2002), particularly regarding themselves and the world (Cromer & Smyth, 2010; Daie-Gabai et al., 2011; Moser et al., 2007), than males. These posttraumatic risk factors have been put forward as potential contributors to sex differences in PTSD (Andrews et al., 2003; Olff et al., 2007). Although negative social support may act as a partial mediator (Andrews et al., 2003), the general conclusion is that it cannot fully account for sex differences in PTSD (Olff et al., 2007). To the best of our knowledge, negative posttraumatic cognitions have not been examined as potential mediators, but one study found that sex still significantly predicted PTSD severity, after posttraumatic cognitions were controlled for (Moser et al., 2007).

## Objective

In summary, the above risk factors cannot fully account for sex differences in PTSD when examined individually. However, if they are combined, the collective influence of these pre-, peri-, and posttraumatic risk factors may explain why females report more PTSD symptoms than males. Whereas few studies have included multiple risk factors in regression analyses (Moser et al., 2007; Spindler et al., 2010), we know of no study that has systematically tested the hypothesis that a combination of these variables will mediate the relationship between sex and PTSD severity. Furthermore, although a regression analysis may suggest that mediation takes place, it is not considered a statistically valid method for testing mediation (Preacher & Hayes, 2008). In the present study, we use the methods of Preacher and Hayes (2008) to test the hypothesis that the combined influence of sexual trauma, neuroticism, depression, anxiety sensitivity, peritraumatic fear, horror, or helplessness, panic, dissociation, and tonic immobility, and posttraumatic cognitions and feeling let down by others will mediate the relationship between sex and PTSD severity.

## Method

### Procedure and participants

The present study is part of a large national cohort questionnaire survey of the psychological impact of bank robberies committed in Denmark from April 2010 to April 2011. A total of 450 employees (73.3%) filled out the first questionnaire a week after the robbery (T_1_; *M=*9.89 days, *SD*=6.30), and 370 participants filled out the second questionnaire 6 months after the robbery (T_2_, *M=*191.7 days, *SD*=13.15; two participants had >20% missing data and were excluded). Thus, the final study population consisted of 368 participants (81.8% of those who participated at T_1_). Only individuals who participated at both time points were included in the present study. Ages ranged from 20 to 65 years (*M=*42.0, *SD=*12.5). A total of 225 participants (61.1%) were female. Participants gave informed consent and were instructed to fill out the questionnaire in relation to the index robbery (see Hansen & Elklit, 2014). The study was in accordance with the Helsinki declaration and approved by the IRB.

### Measures

The questionnaire assessed demographic variables, PTSD severity, pre-, peri-, and posttraumatic factors. PTSD severity was assessed at T_2_. All other variables were assessed at T_1_.

#### PTSD severity

The Harvard Trauma Questionnaire (HTQ part IV) is a self-report scale with a total score indicating PTSD severity and three subscales assessing re-experiencing, avoidance, and arousal (Mollica et al., 1992). Answers are scored on a four-point Likert scale ranging from 1 (not at all) to 4 (all the time). Item scores ≥3 indicate symptom presence. A probable *DSM-IV* PTSD diagnosis is based on the presence of 1 re-experiencing, 3 avoidance, and 2 arousal symptoms (American Psychiatric Association, 2000). The HTQ has shown good internal consistency, test-retest reliability, and concurrent validity (Mollica et al., 1992). The Danish version has been used in diverse trauma populations with reports of good reliability and validity (Bach, 2003). The reliability coefficient for the total score in the present study was excellent (Cronbach's α=0.92).

#### Prior sexual trauma

Participants were asked whether they had experienced sexual abuse or rape. The two items were combined to a dichotomous measure of prior sexual trauma, as no participant reported multiple exposures.

#### Neuroticism

The NEO Personality Inventory-revised (NEO PI-R) short version measures neuroticism on a five-point Likert scale ranging from 0 (strongly disagree) to 4 (strongly agree; Costa & McCrae, 2004). The neuroticism subscale has good psychometric properties, including good reliability (Costa & McCrae, 2004; Rossier, Meyer de Stadelhofen, & Berthoud, 2004). The reliability coefficient in the present study was good (Cronbach's α=0.87).

#### Anxiety sensitivity

The Anxiety Sensitivity Index (ASI) measures beliefs about the harmfulness of anxiety symptoms on a five-point Likert scale ranging from 1 (very little) to 5 (very much; Reiss, Peterson, Gursky, & McNally, 1986). The ASI can be used to generate a total score or three subscale scores reflecting physical, psychological, and social anxiety sensitivity (Stewart et al., 1997). As sex differences have been reported to vary across the three subscales, these were examined individually in the present study. Good test–retest reliability and construct validity have been reported (Reiss et al., 1986). The three subscales had good reliability coefficients in the present study (Cronbach's αs = 0.76–0.87).

#### Depression

The depression subscale from the trait version of the State Trait Anxiety Index (STAI-T; Spielberger, Gorsuch, Lushene, Vagg, & Jacobs, 1983) has been found to be a valid measure of depression with good convergent and discriminant validity (Bados, Gómes-Benito, & Belaguer, 2010). Items are scored on a four-point Likert scale ranging from 1 (almost never) to 4 (almost always). The reliability coefficient in the present study was satisfactory (Cronbach's α = 0.86).

#### Peritraumatic emotional responses

Participants were asked whether they during the robbery had experienced intense fear, horror, or helplessness. The three dichotomous items were combined in a continuous measure of peritraumatic emotional responses equivalent to the *DSM-IV* A_2_ criterion (American Psychiatric Association, 2000). Answers were rated from 0 (none of the responses were reported) to 3 (all three responses were reported).

#### Peritraumatic panic

A modified version of the Physical Reactions Scale (PRS) measured the intensity of peritraumatic panic symptoms (Bryant et al., 2011; Falsetti & Resnick, 1992). Items were rated on a six-point Likert scale ranging from 0 (not at all) to 5 (extremely). There are currently no psychometric data on the PRS (Bryant et al., 2011). The reliability coefficient in the present study was excellent (Cronbach's α=0.91).

#### Peritraumatic dissociation

The Peritraumatic Dissociative Experiences Questionnaire (PDEQ) measures dissociative responses. Items are rated on a five-point Likert scale ranging from 0 (not at all) to 5 (extremely true; Marmar, Weiss, & Metzler, 2004). Good psychometric data has been reported for the PDEQ (Birmes et al., 2005). The reliability coefficient in the present study was good (Cronbach's α=0.87).

#### Tonic immobility

The Tonic Immobility Scale (TIS) originally comprised 10 items assessed on a seven-point Likert scale ranging from 0 (not at all) to 6 (very much; Heidt, Marx, & Forsyth, 2005). However, some of these items assess dissociative and panic-like responses. In order to avoid item overlap with the PDEQ and the PRS, we only assessed the motor aspects of tonic immobility (cf. Birmes et al., 2005; Rocha-Rega et al., 2009). Previous studies report good internal consistency (Birmes et al., 2005). The reliability coefficient in the present study was good (Cronbach's α=0.85).

#### Posttraumatic cognitions

The Posttraumatic Cognition Inventory (PTCI) consists of three subscales assessing negative cognitions about self and the world and self-blame rated on a seven-point Likert scale ranging from 1 (totally disagree) to 7 (totally agree; Foa et al., 1999). Scale scores were calculated for the three subscales. The PTCI has demonstrated high convergent validity with other trauma-related cognition scales (Beck et al., 2004; Foa et al., 1999). The reliability coefficients in the present study were good-to-excellent on all subscales (Cronbach's α=0.70–0.91).

#### Feeling let down

The Crisis Support Scale (CSS) assesses posttraumatic perceived social support (Joseph, Andrews, Williams, & Yule, 1992). Answers are rated on a seven-point Likert scale ranging from 1 (never) to 7 (all the time). The CSS has good reliability and validity (Elklit, Pedersen, & Jind, 2001). Only one item (feeling let down) was relevant in the present study.

### Data analysis

The data was screened for errors and missing values. As there were few missing values (0.3–3.2%), the Expectation Maximisation (EM) algorithm could be used to impute missing data. Mediation analyses were conducted using the Preacher and Hayes (2008) INDIRECT macro for SPSS, using 5000 bootstrap draws. This analysis estimates the path coefficients in a multiple mediator model and generates bootstrap confidence intervals for both total and specific indirect effects of sex on PTSD severity through the potential mediators included in our model. The main focus of the present study is on the total effect, as this allows us to examine whether the combination of risk factors account for the influence of sex on PTSD severity. However, the specific indirect effects may point to which risk factors are especially important in understanding the increased risk of PTSD in females.

## Results

It was estimated that 19 females (8.4%) and 4 males (2.8%) suffered from probable PTSD. In addition, 56 females (24.9%) and 11 males (7.7%) had subclinical PTSD, falling just one avoidance or arousal symptom short of a full diagnosis. In both cases, sex differences were significant (χ^2^=3.84, *p*<0.05 vs. χ^2^=16.23, *p*<0.001).

Females scored higher than males on all measures, except for self-blame and psychological and social anxiety sensitivity (see Table 1). Thus, these two variables were excluded from further analyses. Prior sexual trauma was reported by 2.7% of females and 0.7% of males. Percentages were too low for effects to be reliably detected and thus, sexual trauma was also excluded from further analyses. All other variables correlated significantly with PTSD severity and most of the mediators were also positively associated with each other (see Table 2). In spite of this inter-correlation between variables, there were no problems with multicollinearity (all VIF values <2.7; all tolerance values >0.38), suggesting no content overlap between related concepts.

**Table 1:** Descriptive statistics and sex differences in mediators and PTSD severity

	Females		Males			
			
	M	SD	M	SD	T	Cohen's *d*
PTSD severity	26.77	8.30	22.54	6.60	5.41***	0.564
Neuroticism	14.02	7.98	9.30	6.98	5.80***	0.630
Depression	20.39	5.89	18.10	4.54	4.18***	0.435
Physical anxiety sensitivity	5.30	5.40	3.97	5.01	2.38*	0.319
Psychological anxiety sensitivity	2.37	2.72	1.90	2.52	1.68	0.179
Social anxiety sensitivity	3.11	2.37	3.28	2.34	−0.69	−0.072
Fear, horror, helplessness	1.08	1.00	0.52	0.81	5.83***	0.615
Tonic immobility	8.76	7.25	5.53	5.66	4.77***	0.497
Peritraumatic panic	12.58	12.80	5.41	6.67	7.04***	0.703
Peritraumatic dissociation	18.74	8.07	14.71	4.81	6.00***	0.607
Negative thoughts about self	1.64	0.74	1.35	0.41	4.85***	0.485
Negative thoughts about the world	2.45	1.16	2.07	0.99	3.18**	0.352
Self-blame	1.80	0.95	1.93	1.06	−1.23	−0.129
Feeling let down	2.16	1.83	1.76	1.48	2.26*	0.240

* *p*<0.05

** *p*<0.005

*** *p*<0.001.

**Table 2:** Correlations between pre-, peri- and posttraumatic risk factors and PTSD severity (Pearson's r)

		1	2	3	4	5	6	7	8	9	10	11	12	13
1.	PTSD severity													
2.	Neuroticism	0.45***												
3.	Depression	0.39***	0.71***											
4.	Physical AS	0.33***	0.29***	0.23***										
5.	Psychological AS	0.38***	0.38***	0.39***	0.68***									
6.	Social AS	0.27***	0.18***	0.25***	0.48***	0.43***								
7.	A_2_	0.46***	0.30***	0.26***	0.31***	0.33***	0.16**							
8.	Tonic immobility	0.38***	0.31***	0.30***	0.27***	0.40***	0.23***	0.54***						
9.	Peritraumatic panic	0.51***	0.37***	0.41***	0.43***	0.47***	0.26***	0.63***	0.51***					
10.	Peritraumatic dissociation	0.42***	0.32***	0.32***	0.29***	0.39***	0.28***	0.49***	0.50***	0.64***				
11.	Neg. cog. about self	0.61***	0.58***	0.55***	0.31***	0.48***	0.29***	0.37***	0.38***	0.49***	0.44***			
12.	Neg. cog. about others	0.49***	0.41***	0.37***	0.31***	0.37***	0.28***	0.33***	0.30***	0.42***	0.37***	0.62***		
13.	Self-blame	0.21***	0.11*	0.13*	0.17**	0.17**	0.16**	0.03	0.00	0.16**	0.12*	0.37***	0.35***	
14.	Feeling let down	0.21***	0.20***	0.27***	0.15**	0.21***	0.10	0.18**	0.15**	0.29***	0.17**	0.27***	0.21***	0.06

* *p*<0.05

** *p*<0.01

*** *p*<0.001.

AS: anxiety sensitivity; A_2_: peritraumatic fear, horror, and helplessness; neg. cog.: negative cognitions.

**Fig. 1:**
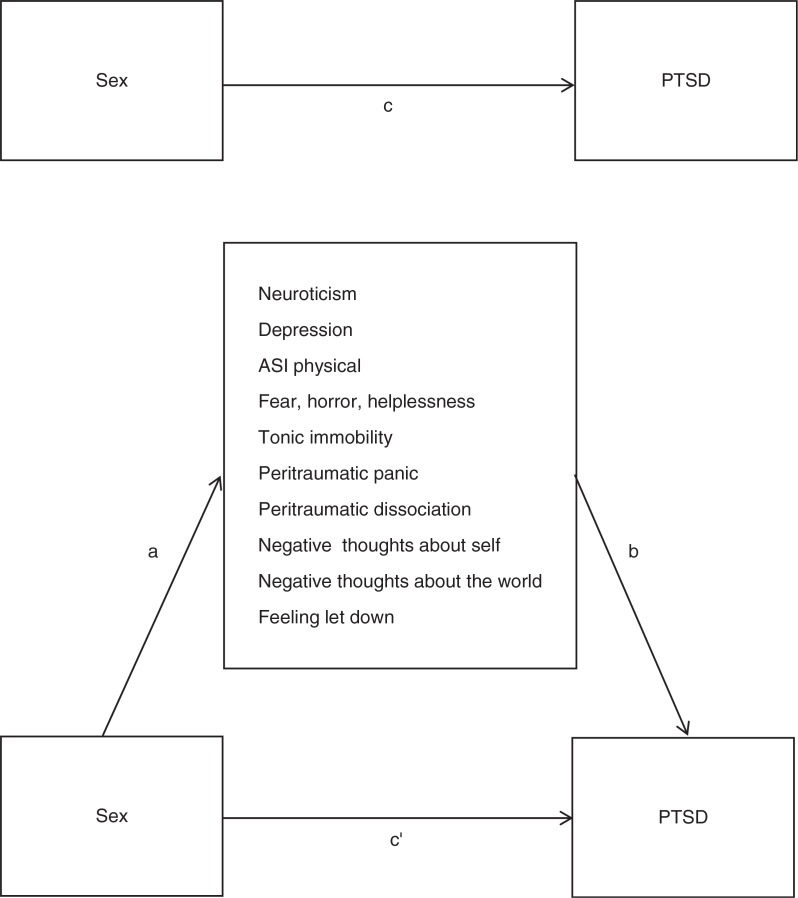
Illustration of a multiple mediation model of sex differences in PTSD. Path c represents the total effect. Path c′ represents the direct effect. Paths a + b represent the indirect effect of sex on PTSD through the mediators. The strength of the mediation is the difference between c and c′. Mediation occurs when path c is statistically significant but becomes non-significant after adjusting for the mediators, resulting in a non-significant path c′.

Only risk factors more prevalent in females were potential mediators. Thus, the variables included in the mediation analysis were neuroticism, depression, physical anxiety sensitivity, peritraumatic fear, horror, and helplessness, tonic immobility, panic, dissociation, negative posttraumatic cognitions about self and the world, and feeling let down. An illustration of the mediation model is shown in Fig. 1.

The mediation analysis shown in Table 3 revealed that the combined risk factors significantly mediated the relationship between sex and PTSD severity. The total effect of sex (c) was −4.1382, *p*<0.0001, suggesting that sex significantly predicted PTSD. However, the direct effect (c′) was −0.7144, *p*=0.30, suggesting that sex no longer predicted PTSD severity when the mediators were taken into account. The total indirect effect had a point estimate of −3.4238 and a 95% BCa CI of −4.6264 to −2.4040, implying that c′ was significantly different from c and that mediation had occurred. Using the formula 1–(c′/c), we calculated that the 10 mediators together accounted for 83% of the total effect. An examination of the specific indirect effects shown in Table 3 indicated that only peritraumatic fear, horror, and helplessness (the A_2_ criterion) as well as negative cognitions about self and the world emerged as uniquely significant mediators. The other variables did not significantly contribute above and beyond these three mediators.

**Table 3:** Mediation analysis of sex differences in PTSD severity: Indirect effects of sex on PTSD severity through the 10 mediators

				Bootstrapping					
				
		Product of coefficients		Percentile 95% CI		BC 95% CI		BCa 95% CI	
		
	Point estimate	SE	Z	Lower	Upper	Lower	Upper	Lower	Upper
Neuroticism	−0.4290	0.2847	−1.5065	−1.0716	0.1499	−1.0964	0.1268	−1.0878	0.1289
Depression	0.1083	0.1890	0.5732	−0.3204	0.5707	−0.2737	0.6191	−0.2747	0.6164
Physical anxiety sensitivity	−0.1081	0.0986	−1.0967	−0.4016	0.0683	−0.4554	0.0440	−0.4556	0.0436
Fear, horror, helplessness	−0.6846	0.2689	−2.5459	−1.3740	−0.0568	−1.4697	−0.1243	−1.5440	−0.1568
Tonic immobility	−0.0866	0.1843	−0.4698	−0.5440	0.3258	−0.5393	0.3296	−0.5196	0.3507
Peritraumatic panic	−0.6429	0.3291	−1.9532	−1.4372	0.1152	−1.4989	0.0694	−1.5470	0.0419
Peritraumatic dissociation	−0.0501	0.2372	−0.2113	−0.6064	0.4945	−0.6139	0.4861	−0.6187	0.4846
Negative thoughts about self	−1.2226	0.3534	−3.4594	−2.1018	−0.4991	−2.1672	−0.5482	−2.2518	−0.5867
Negative thoughts about the world	−0.2931	0.1614	−1.8160	−0.6766	−0.0225	−0.7309	−0.0455	−0.7338	−0.0463
Feeling let down	−0.0151	0.0734	−0.2059	−0.1907	0.1600	−0.2160	0.1377	−0.2173	0.1353
Total	−3.4238	0.6042	−5.6671	−4.5262	−2.3321	−4.6078	−2.3949	−4.6264	−2.4040

## Discussion

To the best of our knowledge, the present study is the first to systematically examine whether a combination of risk factors mediates the relationship between sex and PTSD following a specific traumatic event. We found that the 10 mediators accounted for 83% of the association between sex and PTSD. As almost all of the mediators correlated with each other, only peritraumatic fear, horror, and helplessness and negative posttraumatic cognitions about self and the world added a unique contribution to the relationship between sex and PTSD severity. The finding that peritraumatic fear, horror, and helplessness were among the unique contributors to sex differences in PTSD is in contrast to the argument that peritraumatic variables only affect PTSD indirectly through posttraumatic variables (Lawyer et al., 2006). In contrast, our findings are in accordance with the suggestion by Spindler et al. (2010) that sex differences in the subjective experience and evaluation of the trauma, both during and following the event, are particularly important in explaining sex differences in PTSD. Furthermore, the present findings highlight the importance of posttraumatic cognitions in PTSD in accordance with their inclusion in DSM-5 (American Psychiatric Association, 2013). Although the present findings suggest that sex differences in the remaining risk factors only contribute indirectly to sex differences in PTSD by affecting sex differences in peritraumatic fear, horror, and helplessness and posttraumatic negative cognitions, these results should be interpreted with caution. There was a moderate-to-high degree of correlation between the 10 mediators included in the model. Although these were not strong enough to suggest a problematic overlap of content between the variables, they do suggest that it is the combination of risk factors, rather than the few risk factors found here to have uniquely significant effects, that account for why females are at higher risk of developing PTSD than males. Thus, even though only peritraumatic fear, horror, and helplessness and negative posttraumatic cognitions related to self and the world emerged as unique contributors, we believe that all 10 mediators add to the sex differences in PTSD.

The reasons for the increased prevalence of PTSD symptoms in females may be relevant for treating PTSD. Some of the mediators included in the model presented here are modifiable in therapy. These may include depression, anxiety sensitivity, and especially negative posttraumatic cognitions, the latter of which emerged as unique contributors to sex differences in PTSD. Furthermore, the findings suggest that if we can reduce the negative effects of these mediators, perhaps particularly those of extreme peritraumatic fear, horror, and helplessness and those of posttraumatic negative cognitions, we may be able to reduce sex differences in PTSD. The results in the present study may be of relevance to other trauma populations and to other trauma-related psychiatric diagnoses that are more prevalent in females, such as depression and anxiety.

Although the risk factors included in the present study accounted for most of the association between sex and PTSD, 17% remain unaccounted for. Sexual trauma was not included in the mediation analysis due to low rates. However, sexual trauma was indirectly controlled for, because exposure was too low to have added to the increased PTSD severity in females. In contrast, we have not controlled for betrayal trauma. Females are more likely than males to be exposed to interpersonal trauma perpetrated by someone close to the victim (Goldberg & Freyd, 2006). As betrayal trauma is associated with a particularly high risk of PTSD, it is possible that it may contribute to sex differences. In addition, the study failed to include a number of potential mediators, such as attachment, coping, and gender-role orientation. Furthermore, females may have genetic and biological vulnerabilities, such as a more sensitised HPA axis, that put them at greater risk of developing PTSD compared to males (Moser et al., 2007; Olff et al., 2007). It is possible that these factors may help account for the remaining association between sex and PTSD.

Kistner (2009) has argued that identifying mediators that account for sex differences in psychopathology is not sufficient to explain why males and females differ, if we do not know why sex differences exist in the mediators. In the present study we found that sex differences in 10 pre-, peri-, and posttraumatic risk factors associated with PTSD could account for a large proportion of sex differences in PTSD, but we have not accounted for why females are more likely than males to report these risk factors. Furthermore, whether or not the mediation hypothesis can be confirmed, sex differences in PTSD appear to be much more than the simple question of why females develop more severe PTSD than males. We believe that the role played by sex in PTSD is one of both mediation and moderation. Sex differences in risk factors may account for the increased prevalence of PTSD in females (mediation effects), but at the same time sex differences in the relationship between risk factors and PTSD (moderation effects) may cause males and females to follow different pathways to PTSD (Christiansen & Elklit, 2012). Therefore, if we are to better understand sex differences in PTSD and other psychiatric disorders, more studies must examine risk factors separately in males and females.

On a final note, although many studies claim to examine gender differences in PTSD, most in fact study sex, as only few look into the effects of masculinity or femininity and other gender-related variables on PTSD. Although the terms sex and gender are often used interchangeably, sex refers to the biological distinction between males and females, whereas gender refers to the much more complex cultural understanding of masculine and feminine gender roles embedded in different societies. Whereas knowledge on the impact of sex on PTSD is limited, even less is known about the effects of gender. We believe that the field would benefit from more studies examining the impact of different sex- and gender-related variables on PTSD. Such research may help us better understand exactly how sex and gender affect both PTSD and related individual characteristics, such as the mediators included in the present study.

## Strengths and limitations

The present study has a number of strengths. It is the first of its kind to combine pre-, peri-, and posttraumatic risk factors in a theoretically driven mediation model attempting to explain sex differences in PTSD. Mediation analyses were conducted using an approach that is superior to the methods that have traditionally been used to examine mediation of sex differences in PTSD. Finally, the large population, the equal representation of males and females, and the quasi-prospective design was well suited for conducting mediation analyses.

However, the findings are also subject to a number of limitations. First of all, whereas it is a major strength of the study that peri- and posttraumatic variables were measured soon after the event, thereby reducing interaction with posttraumatic symptom development, it is an important limitation that the supposedly pretraumatic variables were assessed at the same time. As depression, neuroticism, and anxiety sensitivity were assessed approximately 1 week after the robbery they may have been affected by exposure to trauma as well as by acute symptoms of posttraumatic stress. Furthermore, it was not possible to assess PTSD symptoms related to prior traumatic experiences that may have been present prior to the robbery. Therefore, although the pretraumatic variables were considered in the present study to be trait-like characteristics existing prior to the development of PTSD, they may not be fully independent of symptomatology.

Secondly, prior sexual trauma was reported too rarely by participants for this variable to be included in the mediation analyses. One possible explanation for this is that sexual trauma was under-reported in the present study. All data was based on self-report measures that may have resulted in response bias. However, we find it more likely that this particular trauma type is very rare in the population examined in the study. The fact that results were based on a relatively homogenous and high-functioning sample of bank employees exposed to robbery, suggests that they may not be generalisable to other trauma populations.

## Conclusions

The present study examined whether a combination of pre-, peri-, and posttraumatic risk factors could account for why female bank employees report more PTSD than males following a bank robbery. Thus, the purpose of the study was not to predict PTSD, but rather to account for sex differences in PTSD. We found that neuroticism, depression, physical anxiety sensitivity, peritraumatic fear, horror, and helplessness, panic, dissociation, tonic immobility, posttraumatic cognitions about self and the world, and feeling let down together accounted for 83% of the effect of sex on PTSD severity. Only peritraumatic fear, horror, and helplessness and posttraumatic cognitions about self and the world emerged as unique contributors. However, as the mediators were highly correlated with each other, and as models based on only a few risk factors have generally failed to account for sex differences in PTSD, it is our belief that it is the combination of risk factors, rather than the few found here to have a unique effect, that mediate the association between sex and PTSD.

## Supplementary Material

Accounting for sex differences in PTSD: A multi-variable mediation model

Accounting for sex differences in PTSD: A multi-variable mediation model

Accounting for sex differences in PTSD: A multi-variable mediation model

Accounting for sex differences in PTSD: A multi-variable mediation model
