# Supplementary material for: Accounting for sex differences in PTSD: A multi-variable mediation model
Source: Eur J Psychotraumatol. 2015 Jan 19;6:10.3402/ejpt.v6.26068. doi: 10.3402/ejpt.v6.26068 (PMC4300366; doi:10.3402/ejpt.v6.26068)
Supplement: Accounting for sex differences in PTSD: A multi-variable mediation model [file EJPT-6-26068-s001.pdf]

Accounting for sex differences in PTSD: A multi-variable mediation model

Dorte Mølgaard Christiansen, Maj Hansen

Baggrund:

Omtrent dobbelt så mange kvinder som mænd bliver diagnosticeret med posttraumatisk stressforstyrrelse (PTSD). Man ved kun meget lidt om, hvorfor kvinder rapporterer flere PTSD-symptomer, på trods af at mænd generelt udsættes for flere traumer. Dog ved man, at kønsforskelle i PTSD ikke skyldes målefejl, metodologiske bias eller rapporteringsbias men afspejler relle forskelle i mænds og kvinders symptomniveau efter traumer.

Mediationshypotesen foreslår, at kønsforskelle i PTSD er relateret til kønsforskelle i forekomsten af risikofaktorer i tiden før, under og efter traumet. Det betyder, at risikofaktorer, der er mere udbredte blandt kvinder, måske kan forklare kønsforskelle i sværhedsgraden af PTSD. Tidligere studier har generelt fokuseret på få potentielle mediatorer ad gangen og har ofte brugt metoder, der ikke var velegnede til at undersøge mediationseffekter. Disse studier har identificeret en række risikofaktorer, der ser ud til at kunne bidrage til kønsforskelle i PTSD, selvom disse ikke enkeltvis kan forklare kvinders øgede symptomniveau.

Blandt de prætraumatiske variable, der er associeret med en øget forekomst af PTSD, og som oftere rapporteres af kvinder end af mænd, findes tidligere seksuelle traumer, depression, neuroticisme og angstsensitivitet. Med til de vigtigste peritraumatiske risikofaktorer for PTSD hører oplevelsen af intens frygt, hjælpeløshed og rædsel (A2-kriteriet), dissociation og panik under traumet. Alle disse risikofaktorer rapporteres oftere af kvinder end af mænd. Det forventes ligeledes, at kvinder under traumet oplever højere grad af tonisk immobilitet, selvom kønsforskelle i forekomsten af denne risikofaktor for PTSD ikke tidligere er blevet påvist. Endelig kan posttraumatiske risikofaktorer også bidrage til kønsforskelle i PTSD, idet kvinder generelt rapporterer mere negativ social støtte og flere negative kognitioner end mænd.

Formål:

De ovenfornævnte risikofaktorer kan ikke fuldt ud forklare kønsforskelle i PTSD, når de undersøges enkeltvis. Dette studie er det første, der systematisk tester hypotesen om, at en kombination af præ-, peri- og posttraumatiske risikofaktorer, der er mere udbredte blandt kvinder, kan forklare kønsforskelle i sværhedsgraden af PTSD.

Metode:

Studiet er en del af en stor kvasi-prospektiv kohorte af danske bankansatte udsat for røveri i perioden fra april 2010 til april 2011. I alt 450 bankansatte (73,3%) udfyldte en spørgeskemapakke en uge efter røveriet (T1; M = 9,9 dage, SD = 6,3), og 368 deltagere (91,8% af deltagerne ved T_1) udfyldte spørgeskemapakken 6 måneder senere (T_2, M = 191,7 dage, SD = 13,2). PTSD-symptomer blev målt ved T_2, mens øvrige variable blev målt ved T_1. Deltagerne var 20-65 år (M = 42,0, SD = 12,5), og 225 (61,1%) var kvinder. Studiet gør brug af INDIRECT-metoden udviklet af Preacher og Hayes (2008), som er designet til at teste multiple mediationsmodeller ved at udregne bootstrap konfidensintervaller for total og specifikke indirekte effekter.

Resultater:

Som forventet rapporterede kvinder signifikant flere PTSD-symptomer og højere niveauer af neuroticisme, depression, fysisk angstsensitivitet, peritraumatisk frygt, hjælpeløshed og rædsel (A2-kriteriet), tonisk immobilitet, panik, dissociation, negativ social støtte samt negative posttraumatiske kognitioner om sig selv og verden end mænd. Disse variable blev inkluderet i modellen som potentielle mediatorer. Mediationsanalysen viste, at kombinationen af risikofaktorer signifikant medierede forholdet mellem køn og PTSD-sværhedsgrad. Samlet set forklarede de ti

mediatorer 83% af køns indflydelse på PTSD. Særligt peritraumatisk frygt, hjælpeløshed og rædsel samt negative posttraumatiske kognitioner om sig selv og verden fremstod som stærke mediatorer.

Selvom de ti risikofaktorer kunne forklare størstedelen af associationen mellem køn og PTSD, blev de sidste 17% ikke forklaret. Studiet tog højde for mange potentielle mediatorer, men traumer af seksuel karakter blev ikke inkluderet i mediationsanalysen, fordi de blev rapporteret for sjældent. Desuden inkluderede studiet ikke potentielle mediatorer som traumer baseret på svigt (betrayal trauma), tilknytning, coping, kønsrolle-orientering eller genetiske og biologiske faktorer, som kan øge kvinders sårbarhed over for traumer.

Kønsforskelle i PTSD er mere end blot et spørgsmål om, hvorfor kvinder udvikler flere PTSD-symptomer end mænd. Vi argumenterer for, at køn påvirker PTSD gennem både mediation og moderation. Kønsforskelle i risikofaktorer kan forklare den øgede prævalens af PTSD hos kvinder (mediationseffekter), mens kønsforskelle i forholdet mellem risikofaktorer og PTSD (moderationseffekter) kan forårsage, at mænd og kvinder følger forskellige udviklingsveje til PTSD. Hvis vi vil forstå kønsforskelle i PTSD og andre psykiatriske lidelser, bør flere studier undersøge risikofaktorer separat hos mænd og kvinder.

Resultaterne fra dette studie styrkes bl.a. af, at vi har anvendt en metode designet specifikt til at teste multiple mediationsmodeller, samt at studiet er baseret på en stor population med en lige fordeling af kønnene. Derudover er studiet det første af sin slags, der kombinerer præ-, peri- og posttraumatiske risikofaktorer. Det er imidlertid en begrænsning for resultaterne, at de angiveligt prætraumatiske variable blev bedømt efter traumet og dermed kan være blevet påvirket både af selve traumet og af tidlig symptomudvikling.

Konklusion:

Resultaterne tyder på, at kvinder rapporterer flere PTSD-symptomer, fordi de oplever højere grad af associerede risikofaktorer før, under og efter traumet. Vi fandt, at prætraumatisk neuroticisme, depression og fysisk angstsensitivitet, peritraumatisk frygt, hjælpeløshed og rædsel, tonisk immobilitet, panik og dissociation samt posttraumatisk følelse af svigt og negative kognitioner om sig selv og verden kunne forklare 83% af den effekt, køn har på sværhedsgraden af PTSD. Disse resultater tyder på, at man måske kan reducere kønsforskellene i PTSD, hvis man kan reducere de negative effekter af de ti mediatorer, særligt konsekvenserne af peritraumatisk frygt, hjælpeløshed og rædsel samt posttraumatiske negative kognitioner. Resultaterne kan være relevante for andre traumepopulationer og for andre traumarelaterede psykiatriske lidelser, der er mere udbredte blandt kvinder end mænd, såsom depression og angst.

Citation: European Journal of Psychotraumatology 2015, 6: 26068 - http://dx.doi.org/10.3402/ejpt.v6.26068
