# Supplementary material for: Accounting for sex differences in PTSD: A multi-variable mediation model
Source: Eur J Psychotraumatol. 2015 Jan 19;6:10.3402/ejpt.v6.26068. doi: 10.3402/ejpt.v6.26068 (PMC4300366; doi:10.3402/ejpt.v6.26068)
Supplement: Accounting for sex differences in PTSD: A multi-variable mediation model [file EJPT-6-26068-s002.pdf]

# Anteil von Geschlechtsunterschieden in PTBS: Ein multivariables Vorhersagemodell

Dorte Mølgaard Christiansen, Maj Hansen

Hintergrund: Die posttraumatische Belastungsstörung wird doppelt so häufig bei Frauen als bei Männern diagnostiziert. Allerdings ist wenig darüber bekannt, warum Frauen mehr über PTBS -Symptome klagen als Männer. Frühere Studien haben sich in der Regel auf wenige potentielle Mediatoren gerichtet und häufig Methoden verwendet, die nicht ideal sind, um die Vermittlungseffekte zu erheben. Vorhergegangene Studien haben eine Reihe von individuellen Risikofaktoren identifiziert, die wesentlich zu den Geschlechtsunterschieden bezüglich der Schwere der PTBS-Symptomatik beisteuern könnten, auch wenn das nicht die erhöhten Symptomlevel bei Frauen ausreichend erklären kann, wenn diese einzeln untersucht werden.

Ziel: Die vorliegende Studie ist die erste, die systematisch die Hypothese getestet hat, wonach eine Kombination von prä-, peri- und posttraumatischen Risikofaktoren bei Frauen die Geschlechtsunterschiede in der Schwere der PTBS-Symptomatik erklären kann.

Methode: Diese Studie war eine Fragebogenstudie zur Beurteilung von PTBS und verwandten Variablen in 73.3% aller dänischer Bankangestellten die einem Banküberfall innerhalb der Periode von April 2010 bis April 2011 ausgesetzt waren. Die Teilnehmer füllten eine Woche nach dem Überfall (T1, N=450) und sechs Monate nach dem Überfall (T2, N=268; 61.1% Frauen) den Fragebogen aus. Die Auswertung wurde speziell mit einer Analyse entwickelt, um ein multivariables Vorhersagemodell zu testen.

Ergebnisse: Frauen berichteten über mehr PTBS-Symptome als Männer und hatten höhere Werte bei Neurotizismus, Depression, körperliche Angstsensitivität, peritraumatische Angst, Schrecken und Hilflosigkeit (A2-Kriterium), tonische Bewegungslosigkeit, Panik, Dissoziation, posttraumatische negative Erkenntnisse über sich selbst und die Welt, sowie das Gefühl im Stich gelassen zu werden. Diese Variablen wurden in das Modell als mögliche Mediatoren eingeschlossen. Die Kombination von Risikofaktoren vermittelt signifikant die Beziehung zwischen Geschlecht und der Schwere der PTBS-Symptomatik.

Schlussfolgerung: Die Ergebnisse legen nahe, dass Frauen über mehr PTBS-Symptome berichten, weil sie stärker ausgeprägte Risikofaktoren aufweisen. Diese Ergebnisse sind auch für andere Trauma Populationen relevant, sowie für andere traumatisch bedingte psychische Störungen, welche häufiger bei Frauen auftreten, wie beispielsweise Depression und Angst.

Keywords/Schlagwörter: Posttraumatische Belastungsstörung; Geschlechtsunterschiede; multiple Mediatoren; Risikofaktoren; Prädiktoren; Raub; zwischenmenschliche Gewalt

Name of translator: Iris Fischer, Brigitte Lueger-Schuster

Citation: European Journal of Psychotraumatology 2015, 6: 26068 - http://dx.doi.org/10.3402/ejpt.v6.26068
