# Supplementary material for: Accounting for sex differences in PTSD: A multi-variable mediation model
Source: Eur J Psychotraumatol. 2015 Jan 19;6:10.3402/ejpt.v6.26068. doi: 10.3402/ejpt.v6.26068 (PMC4300366; doi:10.3402/ejpt.v6.26068)
Supplement: Accounting for sex differences in PTSD: A multi-variable mediation model [file EJPT-6-26068-s003.pdf]

TSSB'deki cinsiyet farklarını açıklama: Çok değişkenli aracılık modeli

Dorte Mølgaard Christiansen, Maj Hansen

Arkaplan: Neredeyse erkeklerin iki katı kadar sayıda kadın bir TSSB tanısı almaktadır. Ancak, neden kadınların erkeklere oranla daha fazla TSSB belirtisi rapor ettiği hakkında çok az şey bilinmektedir. Önceki çalışmalar genellikle bir kaç tane olası aracı değişkene odaklanmıştır ve sıklıkla aracılık etkilerini test etmek için uygun olmayan yöntemler kullanmışlardır. Tek tek incelendiğinde kadınlardaki artmış belirti seviyelerini açıklamaya yeterli olmasa da önceki araştırmalar TSSB ciddiyetindeki cinsiyet farklarına katkıda bulunabilecek bazı kişisel risk etmenleri belirlemişlerdir.
Amaç: Bu çalışma, kadınlarda daha yaygın olan travma öncesi, sırası ve sonrasındaki risk faktörlerinin birleşimin TSSB ciddiyetindeki cinsiyet farklarını açıklayabileceğine dair savı sistematik bir şekilde test eden ilk araştırmadır.
Yöntem: Çalışma, kısmi-prospektif bir anket araştırması ile Nisan 2010 ve Nisan 2011 arasında banka soygununa maruz kalan Danimarkalı banka çalışanlarının %73,3'ünde TSSB ve ilintili değişkenleri ölçmüştür. Katılımcılar, anketleri soygundan bir hafta (T1, N=450) ve altı ay sonra (T2, N=368; % 61,1 kadın) doldurmuşlardır. Aracılık, özellikle çoklu aracılık modelini test etmek için geliştirilen bir analiz ile incelenmiştir.
Sonuçlar: Kadınlar erkeklere kıyasla daha çok TSSB belirtisi, daha yüksek nevrotiklik, depresyon, fiziksel kaygı hassasiyeti, travma esnası korku, dehşet ve çaresizlik (A2 kriteri), tonik hareketsizlik, panik, disosiyasyon, kendisi ve dünya ile ilgili olumsuz travma sonrası biliş ve hayal kırıklığına uğrama hissi rapor etmişlerdir. Bu değişkenler modele olası aracılar olarak dâhil edilmiştir. Risk etmenlerinin birleşimi, bağlantının %83'ünü açıklayarak cinsiyet ve TSSB ciddiyeti arasındaki ilişkiye anlamlı biçimde aracılık etmiştir.
Tartışma: Bulgular, kadınların daha çok TSSB belirtisi rapor etmesinin nedeninin bağlantılı risk etmenlerini daha yüksek seviyelerde deneyimlemeleri olduğunu ortaya koymuştur. Sonuçlar diğer travma popülasyonları ve depresyon, kaygı gibi travma ile ilişkili diğer psikiyatrik rahatsızlıkların kadınlarda daha yaygın olması ile bağlantılıdır.

Anahtar kelimeler: Travma sonrası stres bozukluğu; cinsiyet farkları; çoklu aracılık; risk etmenleri; yordayıcılar; soygun; kişiler arası şiddet.

Name of translator: Emek Yuce Zeyrek-Rios

Citation: European Journal of Psychotraumatology 2015, 6: 26068 - http://dx.doi.org/10.3402/ejpt.v6.26068
