# Supplementary material for: Accounting for sex differences in PTSD: A multi-variable mediation model
Source: Eur J Psychotraumatol. 2015 Jan 19;6:10.3402/ejpt.v6.26068. doi: 10.3402/ejpt.v6.26068 (PMC4300366; doi:10.3402/ejpt.v6.26068)
Supplement: Accounting for sex differences in PTSD: A multi-variable mediation model [file EJPT-6-26068-s004.pdf]

Accounting for sex differences in PTSD: A multi-variable mediation model

Dorte Mølgaard Christiansen, Maj Hansen

Międzypłciowe różnice w podatności na PTSD: wielozmiennowy model mediacyjny
Wprowadzenie: Średnio dwa razy więcej kobiet w porównaniu do mężczyzn otrzymuje diagnozę PTSD. Jednakże niewiele wiadomo, dlaczego tak się dzieje. Wcześniejsze badania na ten temat wykorzystywały modele mediacyjne identyfikujące czynniki ryzyka odpowiedzialne za powyższy stan, które jednak nie do końca dobrze były przystosowane do badanej problemtyki. Cel: Celem niniejszego badania było przetestowanie hipotezy na temat tego, czy pre- około i potraumatyczne czynniki ryzyka, które wystepują częściej u kobiet, mogą byc odpowiedzialne za różnice międzypłciowe w podatności na PTSD. Metoda: Badanie to miało charakter quasi-prospektywny i kwetionariuszowy i miało na celu okreslenie natęzenia PTSD i powiązanych z tym zaburzeniem czynników ryzyka w grupie 73.3% wszytskich duńskich pracowników banku, którzy eksponowani byli na napady na banki w okresie od kwiestnia 2010 r. do kwietnia 2011 r. Badani wypełniali kwestionariusze 1 tydzień (T1, N=450) oraz 6 miesięcy po napadzie (T2, N=368; 61,11% kobiet). Wykorzystano wielowymairowy model mediacyjny. Wyniki: Wśród kobiet odnotowano wyższe nasilenie PTSD, wyższy poziom neurotycznosci, depresjii, zaabsorbowania lękowego, okołotraumatycznego strachu, poczucia horroru i bezsilności, tonicznego znieruchomienia, paniki, dysocjacji, negatywnych potraumatycznych przekonanń na temat siebie i swiata i poczucia osamotnienia. Wszystkie te zmienne, potraktowane jako mediatory, wyjasniały 83% związku pomiędzy płcią a PTSD. Konkulzje: Wyniki niniejszych badań wskazują, że wsród kobiet notuje się wyższe nasilenie objawów PTSD z uwagi na częstsze wystepowanie u nich różnorakich czynników ryzyka, prowadzących do tego zaburzenia. Wyniki niniejszych badań mogą posłużyć również za próbę wyjaśnienia występowania wyższej częśtości inncyh zaburzeń u kobiet, jak zaburzeń afektywnych i lękowych.

Słwoa kluczowe: zaburzenie po stresie traumatycznym; różnice międzypłciowe; wielozmiennowa mediacja; czynniki ryzyka; predyktory; napad; przemoc interpersonalna.

Name of translator: Marcin Rzeszutek, University of Finance and Management in Warsaw, Poland

Citation: European Journal of Psychotraumatology 2015, 6: 26068 - http://dx.doi.org/10.3402/ejpt.v6.26068
